# Tissue specific stem cell therapy for airway regeneration

**DOI:** 10.1111/cpr.13662

**Published:** 2024-05-27

**Authors:** Dan Bi Park, Jae Yoon Lee, Sung Won Kim, Do Hyun Kim

**Affiliations:** ^1^ Postech‐Catholic Biomedical Engineering Institute, College of Medicine The Catholic University of Korea Seoul South Korea; ^2^ Department of Otolaryngology‐Head and Neck Surgery, Seoul Saint Mary's Hospital, College of Medicine The Catholic University of Korea Seoul South Korea

## Abstract

Secondary atrophic rhinitis (AR), a consequence of mucosal damage during nasal surgeries, significantly impairs patient quality of life. The lack of effective, lasting treatments underscores the need for alternative therapeutic strategies. A major impediment in advancing research is the scarcity of studies focused on secondary AR. Our study addresses this gap by developing an animal model that closely mirrors the histopathological changes observed in patients with secondary AR. These changes include squamous metaplasia, goblet cell hyperplasia, submucosal fibrosis, and glandular atrophy. Upon administering human nasal turbinate stem cells embedded in collagen type I hydrogel in these models, we observed ciliary regeneration. This finding suggests the potential therapeutic benefit of this approach. Our animal models not only emulate the clinical manifestations of secondary AR but also serve as valuable tools for evaluating the efficacy of cell‐based biotechnological interventions.

## Graphical abstract

Tissue specific stem cell therapy on an electrocautery‐induced secondary atrophic rhinitis model.

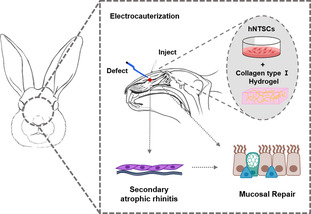

## 1 INTRODUCTION

Turbinate reduction surgery has emerged as a principal therapeutic strategy for the management of chronic hypertrophic rhinitis and allergic rhinitis, substantiated by evidence demonstrating long‐term efficacy.^1^ The performance of this surgical intervention markedly increased from 27,670 procedures in the United States from 2000 to 2015.^2^ Nevertheless, up to 10% of patients undergoing turbinate surgery may develop secondary atrophic rhinitis (AR) due to excessive disruption of the inferior turbinate.^3^

Patients with secondary AR frequently report a paradoxical sensation of nasal obstruction in spite of an anatomically unobstructed nasal passage. The symptomatology is heterogeneous, including impediments in nasal respiration, perception of excessive nasal airflow, dyspnea, compromised pulmonary inflation, and subjective sensations of suffocation.^4^, ^5^, ^6^ This clinical constellation of symptoms has a substantially adverse impact on quality of life and is further exacerbated when concomitant psychological factors are present.^7^, ^8^

Despite various therapeutic endeavours, no intervention has been definitively proven effective in achieving sustained symptom control.^9^ Novel treatment modalities employing cell‐based biotechnological applications are currently under investigation.^10^ The development of a standardized animal model is imperative to assess the efficacy of these emerging therapies. Although a swine model of primary AR has been established, its characteristics are distinct from secondary AR.^11^

We developed an animal model of secondary AR by electrocautery of the nasal turbinate mucosa in New Zealand White rabbits, emulating the procedure performed in human inferior turbinate ablation. Subsequent histological evaluation confirmed the viability of the model. Then the therapeutic potential of human nasal turbinate stem cell (hNTSC) injections with collagen type I hydrogel was assessed.

## 2 RESULTS

### 2.1 Histological changes in secondary AR model

**FIGURE 1:**
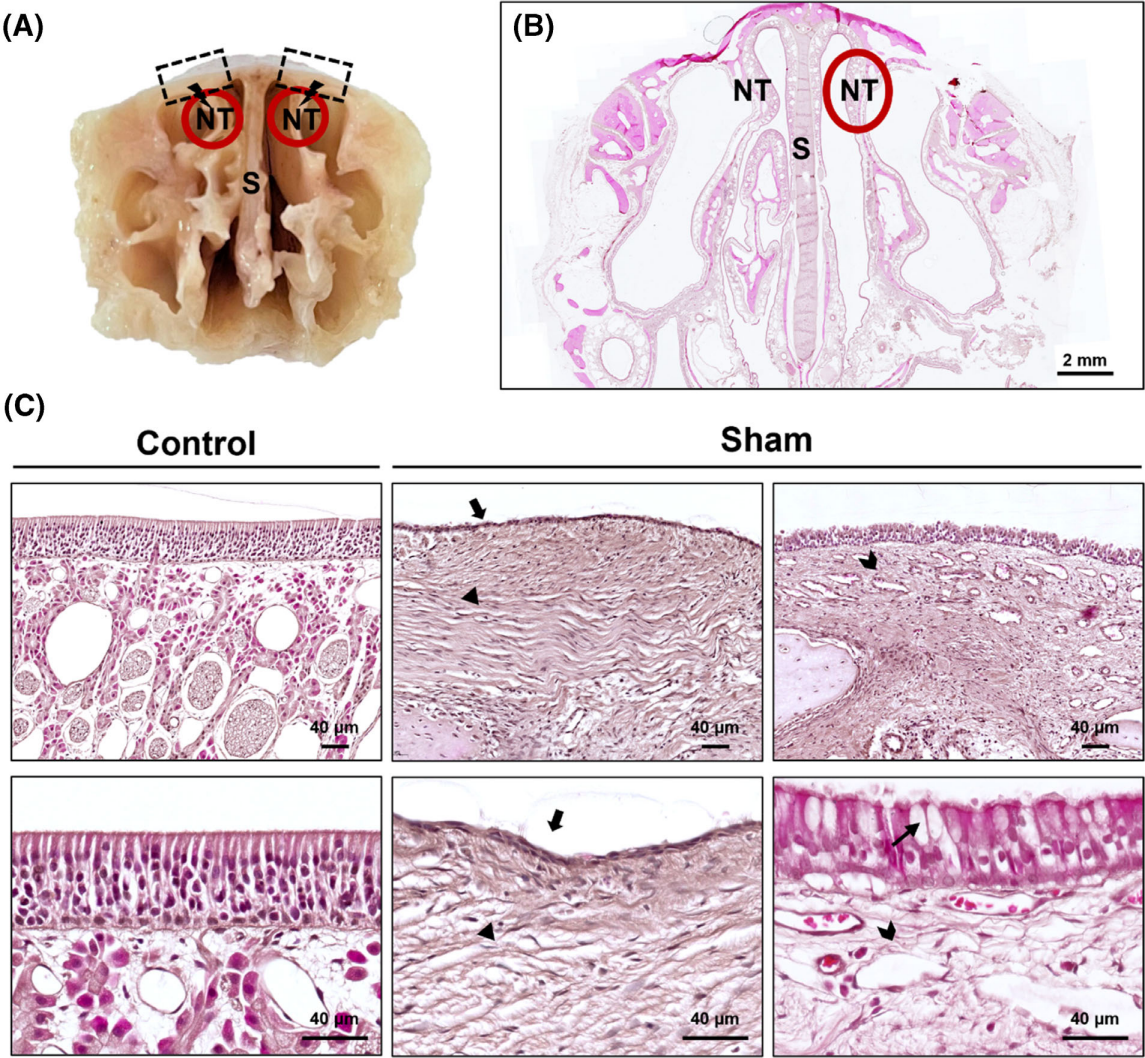
Histological comparisons between control and sham groups. (A) Overview of surgical site. (B) General tissue morphology at 10× magnification. (C) Normal ciliated pseudostratified columnar epithelium in control group (upper panel, 200× magnification; lower panel, 600× magnification). Squamous cell metaplasia (arrow), submucosal fibrosis (arrowhead), glandular atrophy (seagull symbol), and goblet cell metaplasia (thin arrow) in sham group. NT, nasal turbinate; S, nasal septum.

Complete nasal extraction via conical sectioning was performed for model validation (Figure 1A,B). Histological analysis was performed to assess the presence or absence of each histological variables based on the criteria provided in Table 1, which are known to be used for determining each feature.^12^, ^13^, ^14^, ^15^, ^16^ The control group exhibited well‐preserved pseudostratified epithelium with cilia. By contrast, the sham group demonstrated significant histological alterations characteristic of secondary AR, including squamous metaplasia, submucosal fibrosis, glandular atrophy, and goblet cell metaplasia. In areas with markedly prominent submucosal fibrosis, glandular structures were completely diminished (Figure 1C). The comparative assessment of the histological parameters of secondary AR in both groups is summarized in Table 1.

**TABLE 1:** Comparison of histological variables between control and sham groups.

Variables	Criteria	Control (*n* = 3)	Sham (*n* = 3)
Squamous cell metaplasia	Normal <5%^12^	0/3 (0.0)	2/3 (66.7)
Submucosal fibrosis	Normal <20%^13^	0/3 (0.0)	3/3 (100)
Glandular atrophy	Loss of basement membranes of acinic cells to a disappearance of acini^14^	0/3 (0.0)	3/3 (100)
Goblet cell metaplasia	Normal: solid portion <3%^15^	0/3 (0.0)	2/3 (66.7)
Normal ciliated epithelium	Normal: 50%–80% of airway epithelium^16^	3/3 (100)	2/3 (66.7)

*Note*: Data are presented as *n*/*N* (%).

### 2.2 Assessment of characteristics of secondary AR

**FIGURE 2:**
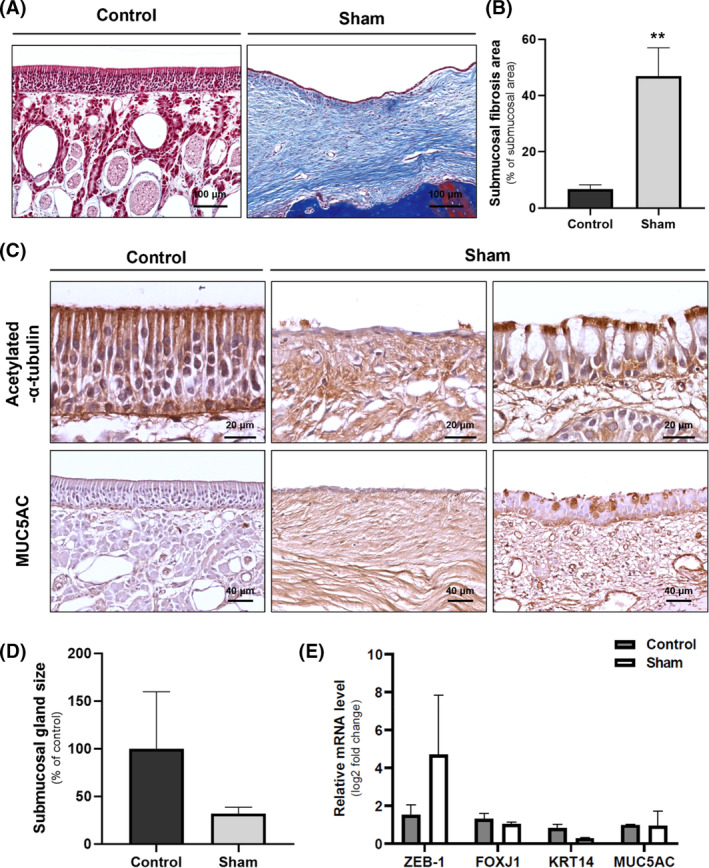
Submucosal and epithelial alterations in secondary AR model. (A) MT staining comparing submucosal fibrosis in control and sham groups at 100×. (B) The percentage of fibrosis in the submucosal area is presented graphically (**p < 0.01). (C) Immunohistochemical staining for acetylated α‐tubulin and MUC5AC. (D) The mean size of submucosal glands was presented as a percentage of the control group. (E) Expression levels of ZEB‐1, FOXJ1, KRT14, and MUC5AC were evaluated using RT‐qPCR. The data are presented as mean ± SD, and no statistical significance was observed between the two groups.

To elucidate submucosal fibrosis, MT staining was performed. The results demonstrated a notable increase in fibrotic cells within the submucosal layer of the sham group compared to the control group (Figure 2A). Subsequently, the proportion of fibrotic structures in the submucosal layer was calculated. The comparison between the control and sham groups revealed a substantial increase, with 6.68% ± 1.18% observed in the control group and 46.99% ± 10.0% in the sham group, demonstrating a significant increase of 40.31%. This difference was statistically significant with a *p*‐value of 0.002. (Figures 2B and S1). IHC was used to identify respiratory epithelial cell markers. Ciliated cells were targeted using antibodies to acetylated α‐tubulin. Although the presence of ciliated cells was sporadic, a considerable reduction in the number of these cells was noted in the sham group. MUC5AC staining revealed two distinct expression patterns in goblet cells. In regions where the pseudostratified columnar structure had deteriorated due to squamous metaplasia, MUC5AC expression was notably absent. However, in areas exhibiting goblet cell metaplasia, MUC5AC expression was focal and concentrated (Figure 2C). To ascertain glandular atrophy, the average size of submucosal glands was compared. It was observed that in the sham group, there was a reduction in gland size to 31.86% ± 6.67% compared to the control group (Figures 2D and S1).

Furthermore, upregulation of the fibrosis marker zinc finger E‐box‐binding homeobox 1 (ZEB1) was observed. With the exception of MUC5AC, markers associated with ciliated pseudostratified columnar epithelium showed low expression as indicated by KRT14 and FOXJ1. (Figure 2E).

### 2.3 Efficacy of cell‐based biotechnological therapeutic agent

**FIGURE 3:**
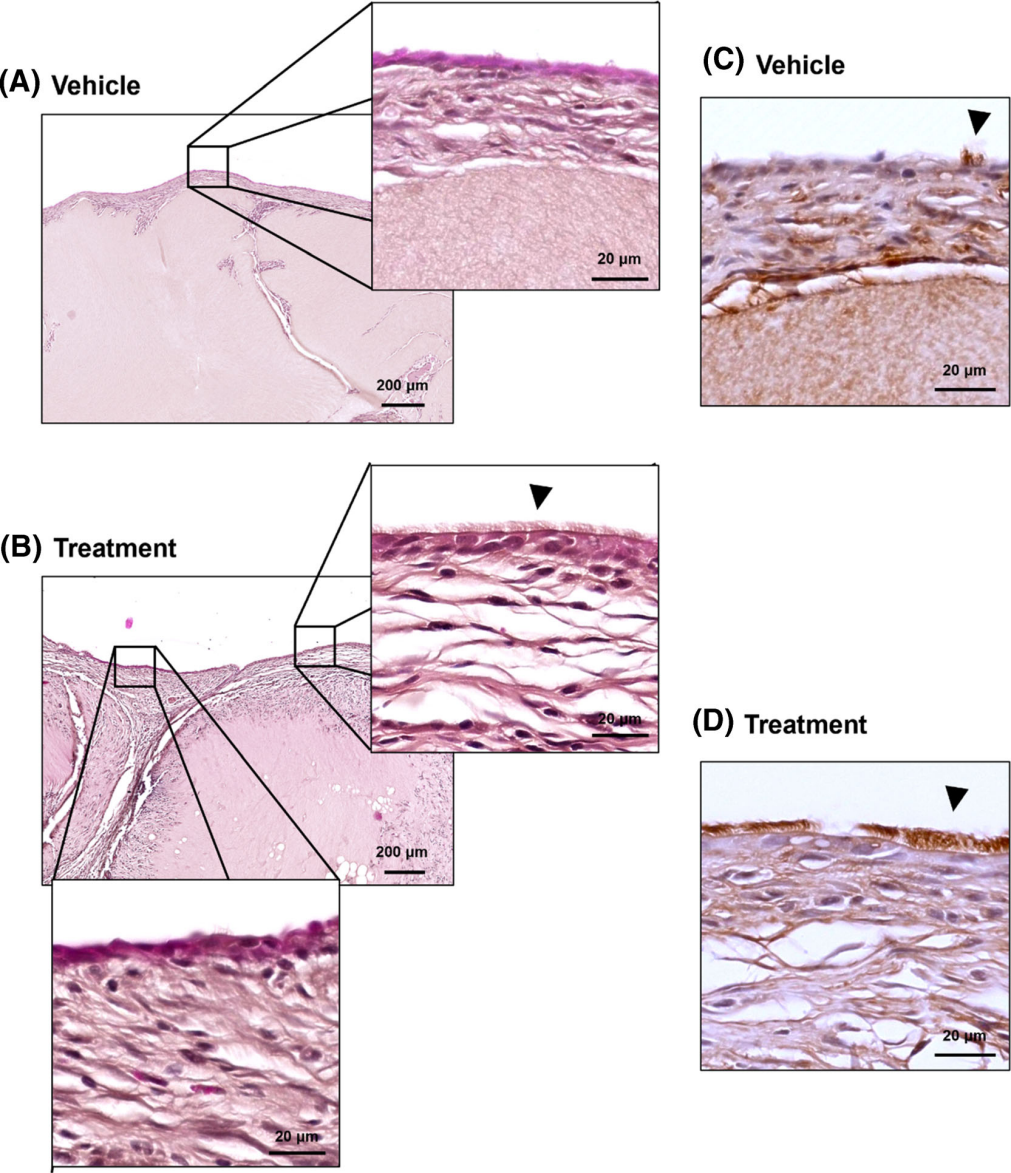
Treatment efficacy assessment in vehicle and treatment groups. (A, B) Comparison of histological features between vehicle and treatment groups (C, D) Immunohistochemical staining for acetylated α‐tubulin. Ciliated cells are indicated by arrowheads.

The treatment group showed ciliary regeneration facilitated by the injected hNTSCs. By contrast, the collagen type I hydrogel showed no therapeutic impact in the vehicle group (Figure 3A,B). The regrowth of cilia at sites of squamous metaplasia was corroborated through staining with acetylated α‐tubulin (Figure 3C,D).

**FIGURE 4:**
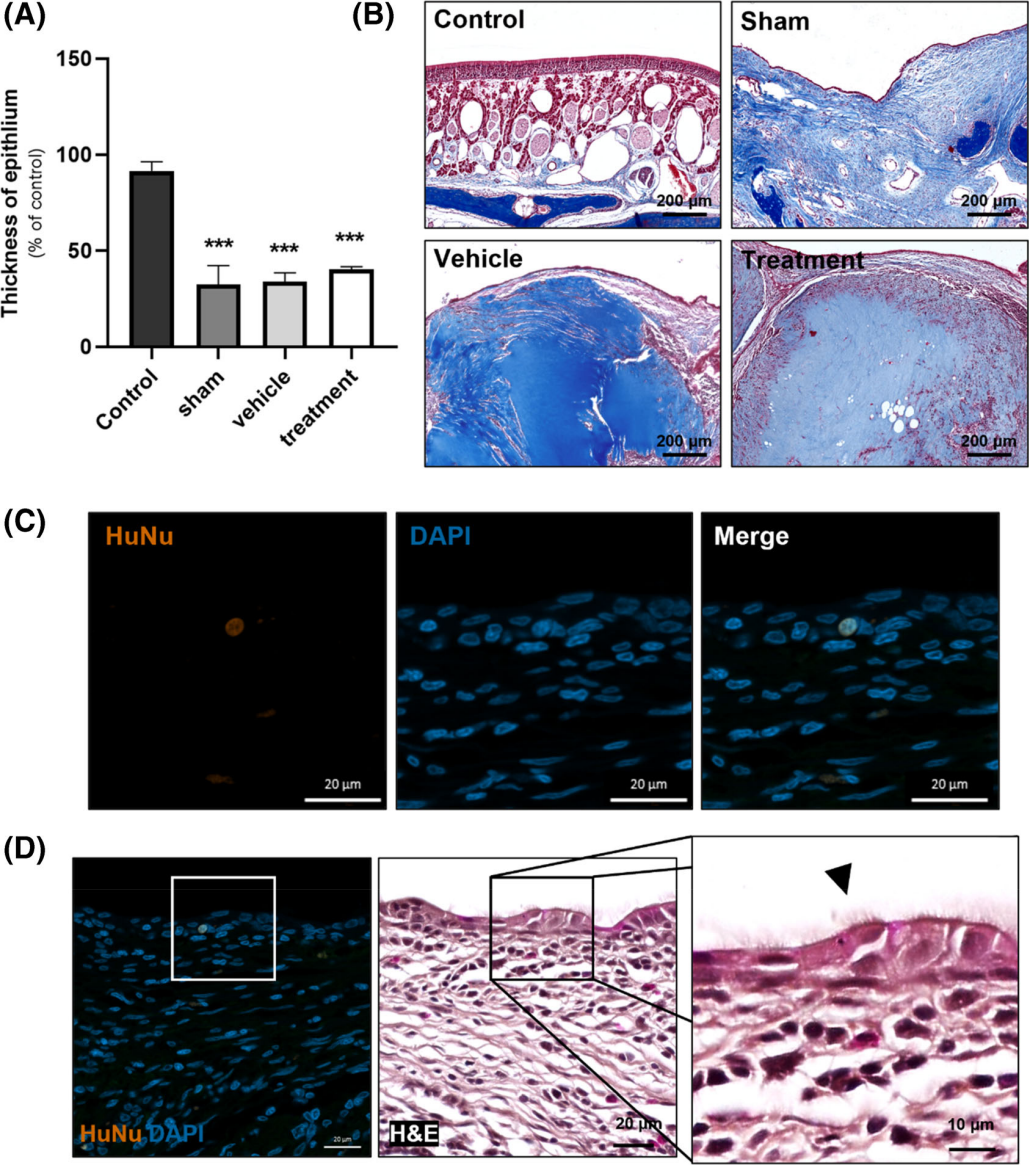
Therapeutic effects of hNTSCs. (A) The comparison of pseudostratified epithelium thickness was presented graphically. (***p < 0.001). (B) MT staining revealing localization of collagen type I hydrogel at the injection site. (C, D) Comparative assessment of therapeutic effects between vehicle and treatment groups. Ciliated cells are indicated by arrowheads.

Using three random H&E images per individual sample, we delineated the thickness of pseudostratified epithelium (Figure S2). The epithelium thickness for each group was obtained in pixel values through ImageJ. These values were then compiled in Table 2 and converted to a percentage based on the first sample in the control group. A notable decrease in the epithelium thickness was observed in the other groups compared to the control group, with mean values ranging from 32.48 ± 17.12 to 40.36 ± 2.55, while the mean value for the control group was 91.62 ± 8.13. Statistical significance was confirmed (control vs sham, vehicle, and treatment; *p* < 0.001), and the findings were graphically represented (Figure 4A). The mean values for the sham and vehicle groups were 32.48 ± 17.12 and 33.91 ± 8.16, respectively, suggesting minimal alterations in epithelium thickness. Conversely, the treatment group exhibited a remarkable elevation to 40.36 ± 2.55 (Table 2).

**TABLE 2:** Pseudostratified epithelium thickness measurement.

Sample no.	Mean ± SD (Sample, *n* = 3)	Mean ± SD (Group, *n* = 3)	*p*‐value
Control 1	100.00 ± 20.54	91.62 ± 8.13	0.0026
Control 2	83.76 ± 8.19		
Control 3	91.11 ± 11.16		
Sham 1	50.28 ± 11.52	32.48 ± 17.12	0.0815
Sham 2	16.13 ± 3.14		
Sham 3	31.02 ± 6.22		
Vehicle 1	31.03 ± 10.98	33.91 ± 8.16	0.0188
Vehicle 2	43.12 ± 5.30		
Vehicle 3	27.58 ± 12.82		
Treatment 1	37.46 ± 5.35	40.36 ± 2.55	0.0013
Treatment 2	41.38 ± 15.70		
Treatment 3	42.25 ± 20.30		

*Note*: Data were normalized from Control 1 mean value to 100.

To verify the efficacy of the treatment group, we first confirmed the presence of hNTSCs at the injection site. MT staining revealed localized subepithelial fibrosis with spindle‐shaped cells in the sham group, while MT staining marked the sites of injected collagen type I hydrogel in the vehicle and treatment groups (Figure 4B). These different sites could be distinguished based on the presence or absence of nuclei as detected by Weigert's haematoxylin. Specifically, cells in sites of exogenous collagen type I hydrogel lacked nuclei, whereas cells in sites of submucosal fibrosis contained nuclei. To distinguish between animal cells and injected hNTSCs, immunofluorescence staining was performed using HuNu, which specifically expresses in human nuclei. HuNu staining revealed the migration of hNTSCs within the epithelial layer at sites of squamous metaplasia, thereby confirming the persistence of injected cells in the treatment group (Figure 4C). Focal regeneration of cilia was also noted adjacent to the HuNu‐localized site (Figure 4D).

## 3 DISCUSSION

The main contributor to secondary AR is turbinate surgery. In one study, 157 of 197 patients with secondary AR had a history of this surgical intervention.^17^ Contrary to the common belief that secondary AR arises from massive resection of the nasal turbinates, the primary cause is actually inferior turbinectomy, and more commonly partial than total turbinectomy.^6^ This indicates that even routine procedures such as partial submucosal electrocautery of the inferior turbinates can lead to secondary AR more frequently than anticipated.

We employed an animal model that successfully emulated the key characteristics of human secondary AR using the same intervention commonly applied in the clinical setting: focal submucosal electrocauterization of the nasal turbinates. The aetiology of this model is fundamentally different from that of previously established swine models of AR, which were induced by toxins from *Pasteurella multocida* and *Bordetella bronchiseptica*.^18^ Significant structural changes in the mucosal tissue have been observed in human patients with secondary AR, including increased squamous metaplasia, submucosal fibrosis, and goblet cell metaplasia as well as a reduced number of submucosal glands.^10^ These histological changes were also present in our novel animal model, implicating its validity for testing the efficacy of therapeutic agents for secondary AR.

The distinct pattern of squamous metaplasia and goblet cell metaplasia observed in human turbinate samples with secondary AR was similarly observed in our model. Although most human patients exhibit squamous metaplasia, 35% also exhibit preserved cilia alongside goblet cells, a feature indicative of a mucosal layer with retained secretory function.^19^ This pattern was congruent with the findings in our animal model. We observed the transformation of normal respiratory epithelium into stratified squamous epithelium, and this transformation was accompanied by glandular atrophy, ciliary loss, and the absent of goblet cells. Such glandular atrophy contributes to nasal cavity dryness, while the loss of cilia hampers the removal of secretions, leading to crust formation. Nevertheless, regions of goblet cell metaplasia with preserved ciliated mucosa were also identified. This is likely a compensatory shift from ciliated cells to goblet cells in response to chronic epithelial irritation. Another study supports this view, suggesting that these changes are not the result of goblet cell hyperplasia but rather represent a transformation of ciliated cells triggered by surgical damage.^20^

We confirmed these changes through both tissue histology and identification of molecular markers representing the characteristics of altered tissues. Markers of ciliated respiratory cells, namely acetylated α‐tubulin and FOXJ1, showed low expression at both the protein and mRNA levels. Conversely, the secretory cell marker MUC5AC remained at a similar level in both the sham and control groups, suggesting a compensatory increase in mucinous glands due to goblet cell metaplasia. Diffuse fibrosis was assessed using MT staining, which visualizes collagen fibres in blue, as a high concentration of collagen is localized within the fibrotic cells. In all subjects that underwent electrocauterization, fibrosis in the submucosal layer was observed to be blue and appear spindle‐shaped, indicating an extensive area. Furthermore, we observed an elevation in the mRNA expression of ZEB1, a key contributor to fibrosis occurrence.

The challenge in treating secondary AR lies in the absence of known definitive treatment agents. Patients experience persistent, debilitating symptoms that adversely affect their quality of life and are often limited to only conservative management options such as saline sprays, lubricants, and antidepressants, which offer only temporary relief and fail to reverse tissue‐level changes.^9^ Various materials and surgical techniques have been proposed to reduce the size of the nasal passages, simulating the nasal conditions prior to surgical intervention or injury. This reduction aids in the heating and humidification of air during inhalation.^21^ Many materials, ranging from injectable substances such as carboxymethylcellulose/glycerin gel to allografts, xenografts, and synthetic substances, have demonstrated favourable effects regardless of the material type.^22^ The selection of suitable materials requires consideration of several factors, such as the risk of foreign body reaction, protrusion, and infection for synthetic materials and potential donor‐site morbidity for autologous costal cartilage harvesting.^23^

Emerging regenerative approaches using materials such as aqueous extract of human placenta, stromal vascular fraction, and platelet‐rich plasma have shown promise.^24^, ^25^, ^26^, ^27^ Recent investigations into the synergistic application of stem cells with bioactive materials for tissue engineering are encouraging, but they necessitate rigorous animal model testing for efficacy and safety.^10^

The model established in the present study served as a platform for evaluating cell‐based biotechnology treatments. We employed hNTSCs in conjunction with collagen type I hydrogel, which exhibited tissue regenerative capabilities in previous studies.^28^, ^29^, ^30^ Additionally, synergy in cell migration, viability, and proliferation was observed when hNTSCs were combined with collagen type I hydrogel, corroborating earlier findings.^31^, ^32^, ^33^ To assess the therapeutic impact, two treatment groups were established: one received only collagen type I hydrogel, while the other received a combination of the hydrogel and hNTSCs. To verify the presence of injected collagen at the electrocauterization site, MT staining was performed. In the treatment group, unlike the vehicle group, cells were observed due to the staining of nuclei inside collagen by Weigert's haematoxylin. To confirm the identity of these cells as hNTSCs, immunofluorescence with HuNu was performed. Even 2 months after administration, the persistence of hNTSCs was indicated, with expression observed towards the epithelium side, suggesting migration in that direction. Consequently, ciliary regeneration was observed in the region of hNTSCs, suggesting their potential influence on regeneration. However, further research is needed on this point.

The most important histological change in the secondary atrophic rhinitis model is squamous cell metaplasia. This change contributes major symptoms of atrophic rhinitis such as dryness of the nasal mucosa, crust formation, and infection. Therefore, the most important point in terms of treatment effectiveness is the conversion of squamous cell metaplasia epithelium back into ciliated epithelium. Unlike the sham group, we were able to confirm that the ciliated epithelium was regenerated in the area where stem cells were injected.

The exact pathways by which stem cells facilitate tissue regeneration remain unclear. Research has demonstrated the potential of human neural stem cells and human umbilical cord mesenchymal stem cells to differentiate into various neural lineage cells and osteocytes.^34^, ^35^, ^36^ Moreover, evidence suggests that stem cells can induce tissue restoration via paracrine signalling.^37^, ^38^ Although the specific mechanism underlying the regenerative effects observed in our therapeutic model remains unclear, our animal model provides a promising basis for future investigations into the efficacy of diverse treatment strategies.

We made a secondary atrophic rhinitis animal model and investigated the therapeutic potential of stem cells, but this study has several limitations. First, we performed injection immediately after model creation. This protocol was considered because surgery to open the rabbit's nasal cavity after anaesthesia may increase the rabbit's morbidity. Although relatively time is required to model drug administration, infection, and inflammation induction, this model was able to be produced because tissue changes due to electrocauterization occur relatively quickly. Second, this study confirmed the model creation and treatment effect of stem cells at a single time point. However, studying histological changes at different time points after treatment would also provide meaningful information. Additional research would be needed on this point in the future. Third, stem cell treatment and effectiveness verification should be conducted together with evaluation of not only histological changes but also symptom improvement. However, there were limitations to performing this in animal models. Further research will need to be done in the future on the therapeutic effects and evaluation methods of stem cells.

## 4 MATERIALS AND METHODS

### 4.1 Animal studies

Specific‐pathogen‐free New Zealand white rabbits (male, 3–3.5 kg body weight) were purchased from Kangda (Qingdao, China). All procedures adhered to relevant ethical guidelines and were approved by the Institutional Animal Care and Use Committee of the Catholic University of Korea (Approval No. CUMS‐2022‐0289‐03).

### 4.2 Isolation and culture of hNTSCs from human turbinate tissue

**FIGURE 5:**
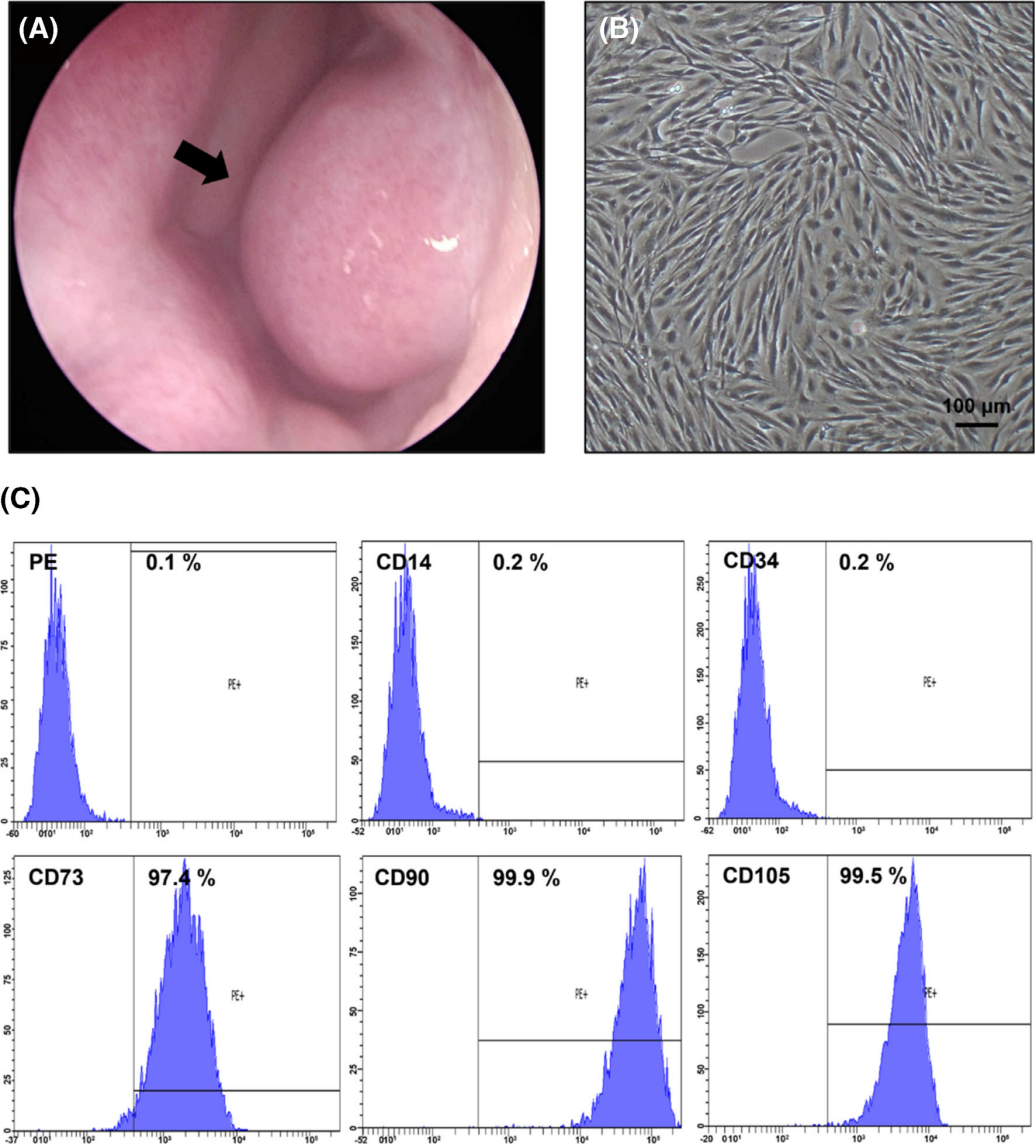
Isolation, culture, and characterization of hNTSCs. (A) Endoscopic image of donor's inferior turbinate (arrow). (B) Cultured hNTSCs isolated from turbinate tissue at 40× magnification. (C) Confirmation of hNTSC surface markers (IgG‐PE, CD14, CD34, CD73, CD90, and CD105).

Fresh hNTSCs were sampled from the inferior turbinate tissue of a 22‐year‐old man (Figure 5A). The procedure was conducted at Seoul Saint Mary's Hospital and received ethical approval from the Institutional Review Board of the Catholic University of Korea (Approval No. KC18TESI0167). The tissue was initially rinsed with a gentamicin solution in the surgical suite; it was then washed with an antibiotic–antimycotic solution (Gibco, Gaithersburg, MD, USA) and Dulbecco's phosphate‐buffered saline (PBS) (Welgene, Namcheon, South Korea).

Next, the tissue was sectioned into fragments with a thickness of 0.5 mm and incubated at 37°C in Dulbecco's Modified Eagle Medium (Gibco) supplemented with 10% fetal bovine serum (Gibco) and Minimum Essential Medium‐α (Gibco). The culture environment was maintained at 5% carbon dioxide and sealed with a sterilized glass slide.^39^

After a 2‐week cultivation period, the cells were harvested from the plate and dissociated using a 1‐mL solution of 0.25% Trypsin and 1 mM ethylenediaminetetraacetic acid. The morphology and concentration of cells were examined under 40× magnification (Figure 5B).

### 4.3 Flow cytometry analysis

A single‐cell suspension of hNTSCs was prepared and incubated for 1 h with phycoerythrin‐conjugated antibodies specifically targeting mesenchymal stem cell surface markers, including CD14, CD34, CD73, CD90, and CD105 (BD Pharmingen, San Diego, CA, USA). Then the cells were washed and resuspended in Dulbecco's PBS. Flow cytometry analysis (BD FACSCanto II; Becton Dickinson, Franklin Lakes, NJ, USA) was performed to assess the constituent cell populations. The hNTSCs were selected based on their confirmed negative expression for the haematopoietic cell markers CD14 and CD34 coupled with their positive expression for the mesenchymal stem cell markers CD73, CD90, and CD105 (Figure 5C).

### 4.4 Preparation of the treatment agent

To formulate the therapeutic agent administered to the rabbits in the treatment group, hNTSCs were harvested from the culture dish and pelleted. Then these cells were amalgamated with a cold collagen type I hydrogel (COLTRIX® TendoRegen; Ubiosis, Sungnam, South Korea) at a concentration of 2 × 10^6^ cells/mL. A 1 mL insulin syringe with a 29‐gauge needle (BD Pharmingen) was used to accurately administer 100 μL aliquots of the agent. In the vehicle group, collagen type I hydrogel was dispensed in 100 μL aliquots to ensure uniformity of the administration procedure.

### 4.5 Surgical procedure

**FIGURE 6:**
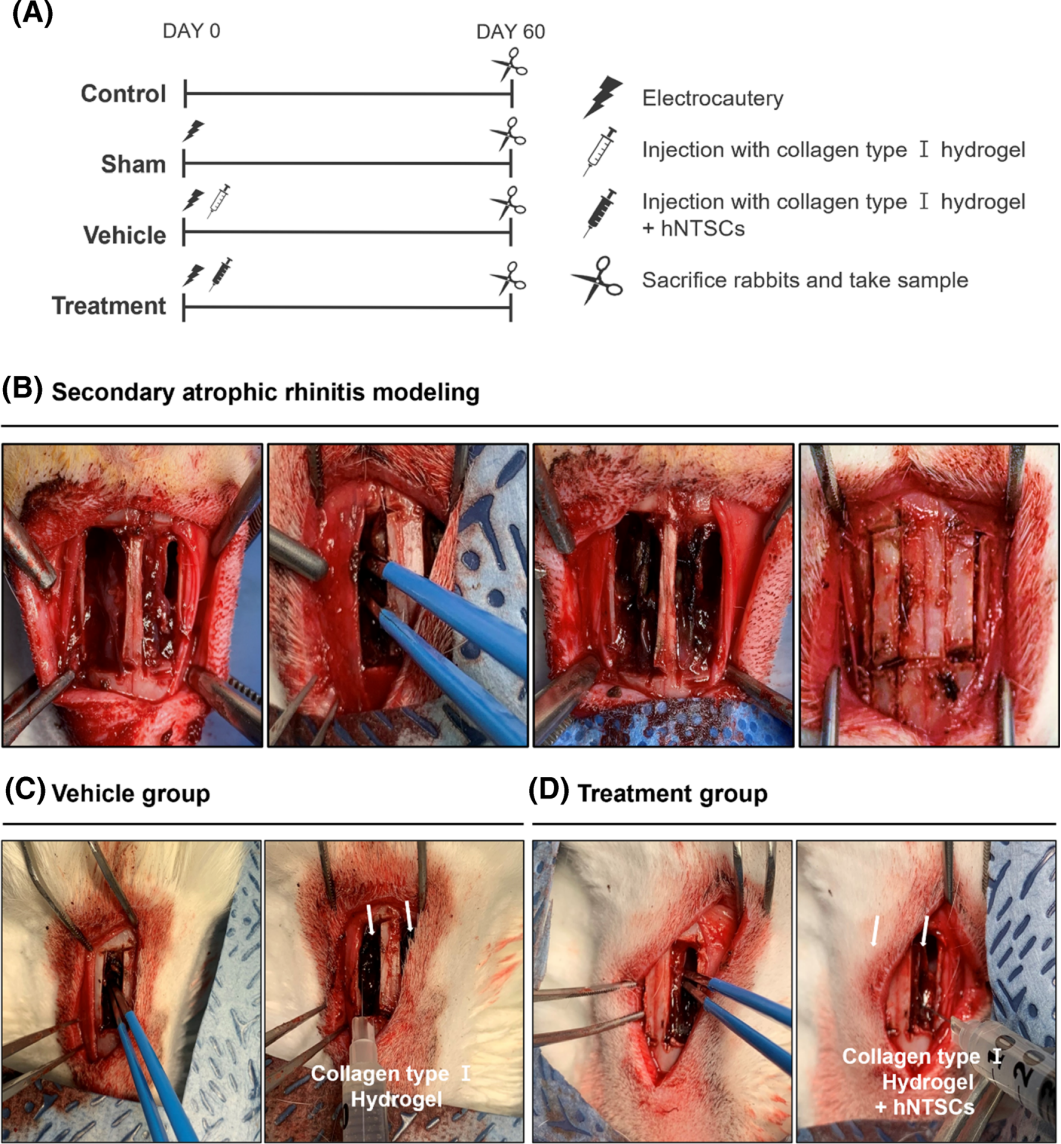
Induction and treatment of secondary AR in rabbit models. (A) Control and experimental groups. (B) Electrocautery‐based induction of secondary AR to establish the sham group. (C) Collagen type I hydrogel injection in vehicle group. (D) Administration of a composite of hNTSCs and collagen type I hydrogel in the treatment group.

Rabbits (*n* = 3 in each group) were randomly divided into four groups: a sham group that underwent turbinate surgery, a vehicle group that underwent turbinate surgery and intraoperative collagen hydrogel injection, a treatment group that underwent turbinate surgery and hNTSC application, and a control group that underwent no intervention. (Figure 6A). In the vehicle and treatment groups, injection was performed immediately after turbine electrocauterization and bone reposition was performed.

To explain in detail the model production method, an initial midline incision extending approximately 3 cm from the nasal tip was made along the nasal dorsum. After elevation of both skin and periosteal flaps, bone flaps measuring 2.5 × 0.5 cm^2^ were excised on both sides of the septum using a micro‐motor handpiece (Saeshin, Daegu, Korea). These bone flaps were subsequently preserved in a sterile saline solution (Figure 6B). The nasal turbinates were electrocauterized in the sham, vehicle and treatment groups using a bipolar device. To evaluate the therapeutic efficacy, a 100 μL aliquot of the formulated hNTSC treatment agent was administered to the nasal turbinates of the rabbits in the treatment group. Conversely, an equal volume of collagen type I hydrogel was administered in the vehicle group (Figure 6C, D). The bone flaps were repositioned to their original anatomical sites, and the skin and periosteal flaps were sutured. All surgical procedures were performed by a single rhinology specialist. All samples (*n* = 12) were obtained 2 months after turbinate surgery. Among 9 rabbits underwent surgery, there were no severe adverse events or deaths related to surgery during the study period.

### 4.6 Tissue processing

The rabbits were euthanized with potassium chloride. The samples were fixed overnight in 4% formaldehyde and rinsed in PBS. The samples were decalcified in 1% ethylenediaminetetraacetic acid (pH 8) for 2 months and then embedded in paraffin.

### 4.7 Histological analysis

Sections of 4.5 μm thickness were deparaffinized in xylene and rehydrated through a graded ethanol series. After deparaffinization, the sections were stained with Mayer's Haematoxylin (Abcam, England) and Eosin (Daejung, Korea). To measure the pseudostratified epithelium thickness, stained sections were captured randomly from three different fields for analysis under a ×400 magnification. The area of interest for measuring pseudostratified epithelium thickness was specified, and the pixel values for area and width were obtained using ImageJ (https://imagej.net/ij/). The area value was divided by the width value to derive the length value (pixels). Other sections were stained using the Masson's trichrome (MT) method according to the manufacturer's protocol (Abcam).

### 4.8 Immunohistochemistry (IHC) and immunofluorescence (IF) analysis

All sections underwent 5‐minute epitope retrieval in 0.01 M citrate buffer (Sigma‐Aldrich, St. Louis, MO, USA). Endogenous peroxidase activity was neutralized with 3% hydrogen peroxide. The sections were blocked with 5% normal goat serum (Vector Laboratories, Burlingame, CA, USA) for 1 h.

For the immunohistochemical analysis, the following two primary antibodies were applied for a 2 h incubation period: anti‐MUC5AC (1:100) (Abcam) and anti‐acetylated α‐tubulin (1:100) (Santa Cruz Biotechnology, Dallas, TX, USA). The sections were washed with PBS and treated with a mixture of anti‐rabbit and anti‐mouse horseradish peroxidase polymer (GBI Labs, Bothell, WA, USA) for 30 min. Diaminobenzidine (GBI Labs) staining and haematoxylin counterstaining followed.

For the immunofluorescence analysis, the sections were incubated with a primary anti‐human nuclei (anti‐HuNu) antibody (1:200) (Sigma‐Aldrich) for 2 h, washed, and treated with a secondary goat anti‐mouse IgG Alexa Fluor™ 555 antibody (1:1000) (Invitrogen, Carlsbad, CA, USA) for 1 h. DAPI (1 μg/mL) (Invitrogen) counterstaining was then performed. The slides were examined using a confocal laser microscope (LSM 800; Carl Zeiss, Oberkochen, Germany).

### 4.9 RNA extraction and real‐time quantitative polymerase chain reaction

Total RNA was extracted from unfixed tissue using TRIzol (Thermo Fisher Scientific, Waltham, MA, USA). Reverse transcription was conducted with a cDNA synthesis kit (iScript™; Bio‐Rad, Hercules, CA, USA), followed by amplification on a polymerase chain reaction detection system (CFX96™; Bio‐Rad). Relative mRNA levels were calculated using the 2^−ΔΔCt^ method, with HPRT1 as a control. The primers are listed in Table 3.

**TABLE 3:** Primer sequences.

Gene	Primer	Sequence
HPRT1	Forward	GAC CAG TCA ACA GGG GAC AT
	Reverse	CTT GCG ACC TTG ACC ATC TT
FOXJ1	Forward	TCG ACT GGG AAG CCA TCT
	Reverse	GTC GAA GTC CAG GCT GTT G
Keratin 14	Forward	GAA GGA GGA ACT GGC CTA CC
	Reverse	TCT CGT ACT GGT CAC GCA TC
MUC5AC	Forward	TCT GCT GTC CCG AGA GAA CAC
	Reverse	CTG CCA TCA CAA ATG ACC AC
ZEB1	Forward	GCC TAC AGA ACC CAA CTG GA
	Reverse	TCT CTC CGC TGT GAA TCC TT

### 4.10 Statistical analysis

The statistical analyses were conducted using a two‐way ANOVA or t‐test. The analyses were performed using Prism 8.0 software (GraphPad Software, San Diego, CA, USA).

## AUTHOR CONTRIBUTIONS

DBP and JYL contributed equally to this work as first authors. SWK and DHK contributed equally to this work as corresponding authors. DBP conducted the primary experiments, managed the experimental data, and wrote a brief draft of the manuscript. JYL validated the experimental results, equally contributed to writing the initial draft, and completed the manuscript. DHK investigated the study design, developed the experimental methods, validated the results, and edited the manuscript. SWK supervised the project execution, managed administrative tasks, and contributed to the manuscript review. All authors reviewed and approved the final manuscript.

## FUNDING INFORMATION

This work was supported by National Research Foundation of Korea grants funded by the Korean government (Ministry of Science and ICT) (No. RS‐2023‐00209494) and a Korean Fund for Regenerative Medicine grant funded by the Korean government (No. 23C0121L1). The sponsors had no role in the study design, data collection and analysis, decision to publish, or preparation of the manuscript.

## CONFLICT OF INTEREST STATEMENT

The authors declare that they have no competing interests, financial or otherwise, that could influence the work reported in this manuscript.

## Supporting information


**Data S1.** Supporting Information.

## Data Availability

The data that support the findings of this study are available from the corresponding author upon reasonable request.
