# Supplementary material for: Tissue specific stem cell therapy for airway regeneration
Source: Cell Prolif. 2024 May 27;57(10):e13662. doi: 10.1111/cpr.13662 (PMC11471432; doi:10.1111/cpr.13662)
Supplement: Supplementary file 1 — Data S1. Supporting Information. [file CPR-57-e13662-s001.docx]

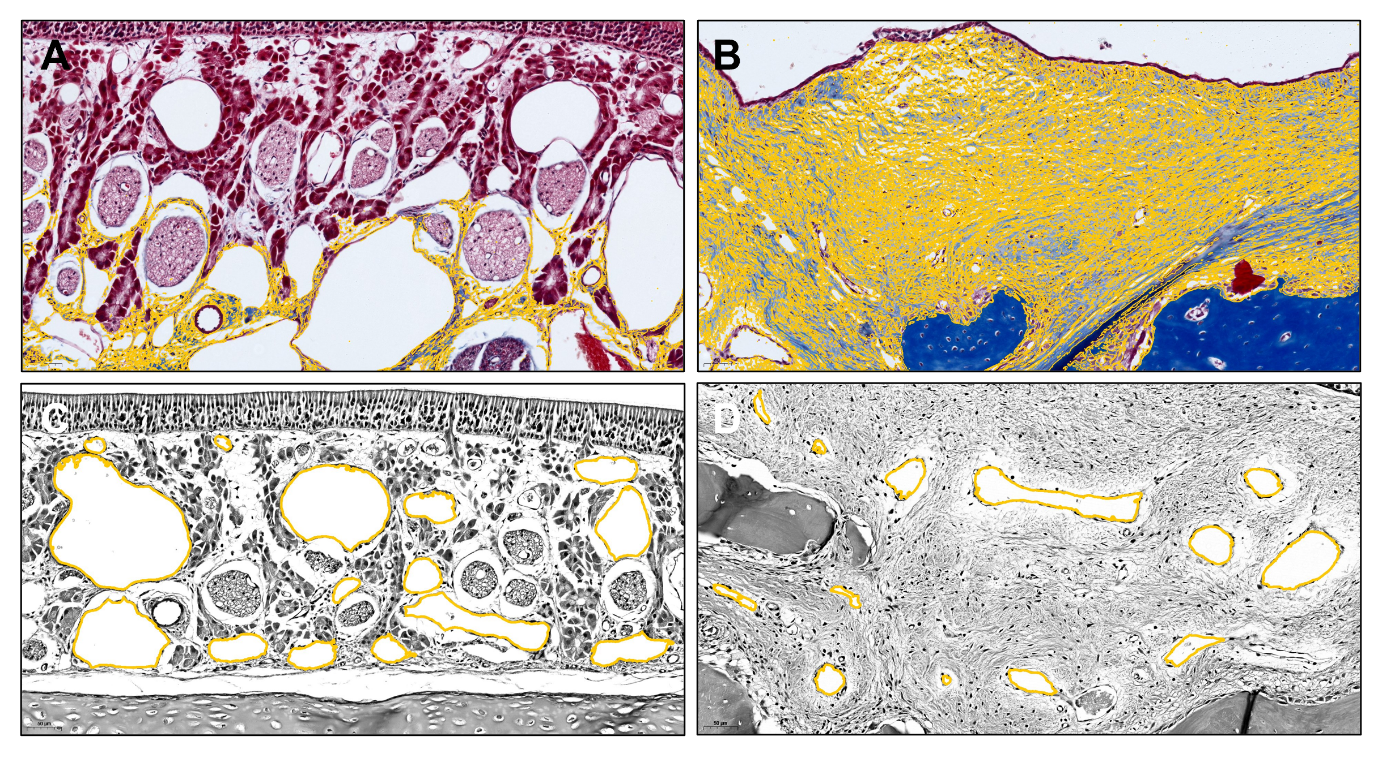


**Figure S1. Area selected for measuring altered characteristics in secondary AR**

Submucosal fibrosis and gland size were measured by outlining the area in yellow. Measurements were conducted using images at 200x magnification. The upper images depict MT staining for fibrosis assessment, while the lower images show H&E staining with grayscale processing to facilitate submucosal gland area selection. (A, C) Control, (B, D) Sham group.

**
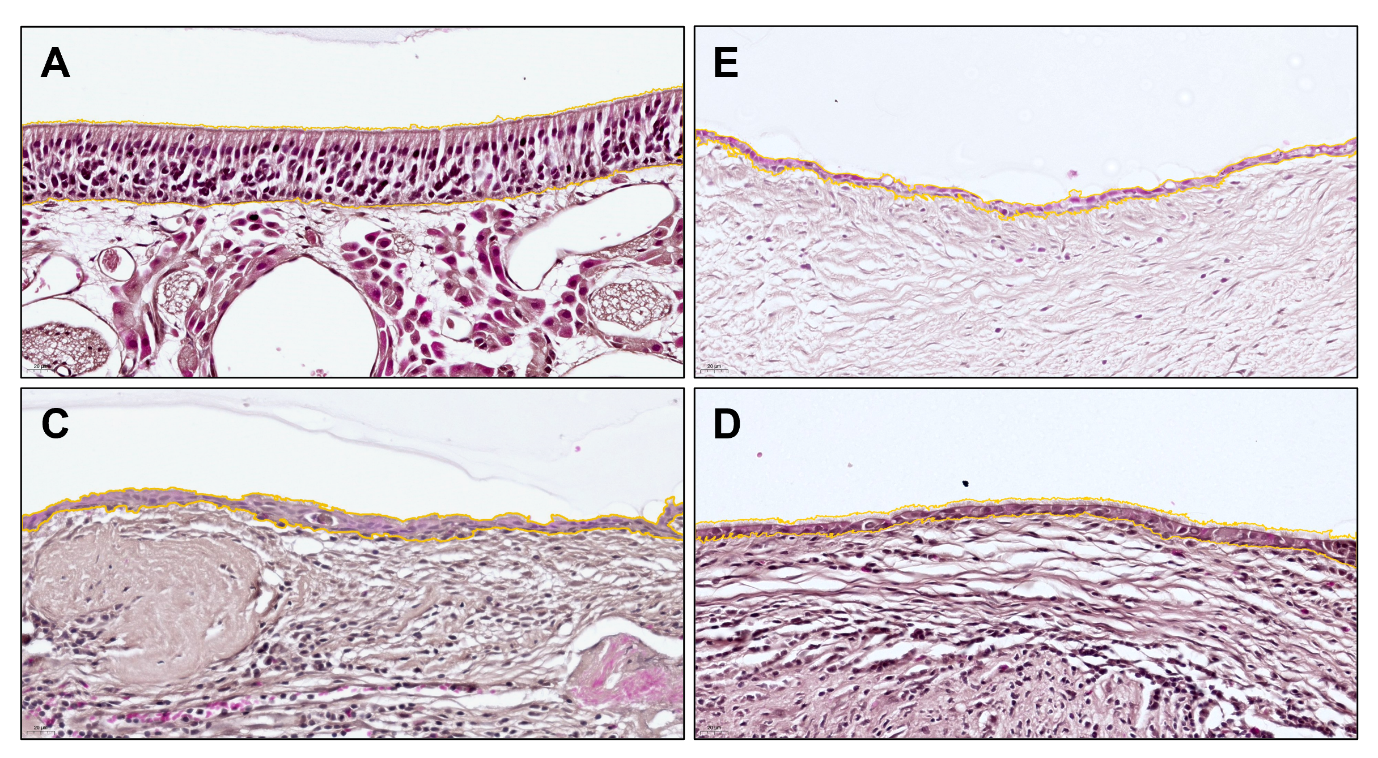
**

**Figure S2. Area selected for measuring altered characteristics**

To measure pseudostratified epithelium thickness, H&E images used at 400x magnification were utilized. The area of pseudostratified epithelium was selected and measured for analysis. (A, C) Control, (B, D) Sham, (E) Vehicle, and (F) Treatment group.
